# Artificial intelligence for aging research in cancer drug development

**DOI:** 10.18632/aging.204914

**Published:** 2023-11-18

**Authors:** Dorsa Shirini, Lawrence H. Schwartz, Laurent Dercle

**Affiliations:** 1 Department of Radiology, Columbia University Medical Center, New York, NY 10032, USA; 2 Department of Radiology, Memorial Sloan Kettering Cancer Center, New York, NY10032, USA; 3 Shahid Beheshti University of Medical Sciences, Tehran, Iran

**Keywords:** artificial intelligence, aging, cancer research

**Figure 1:**
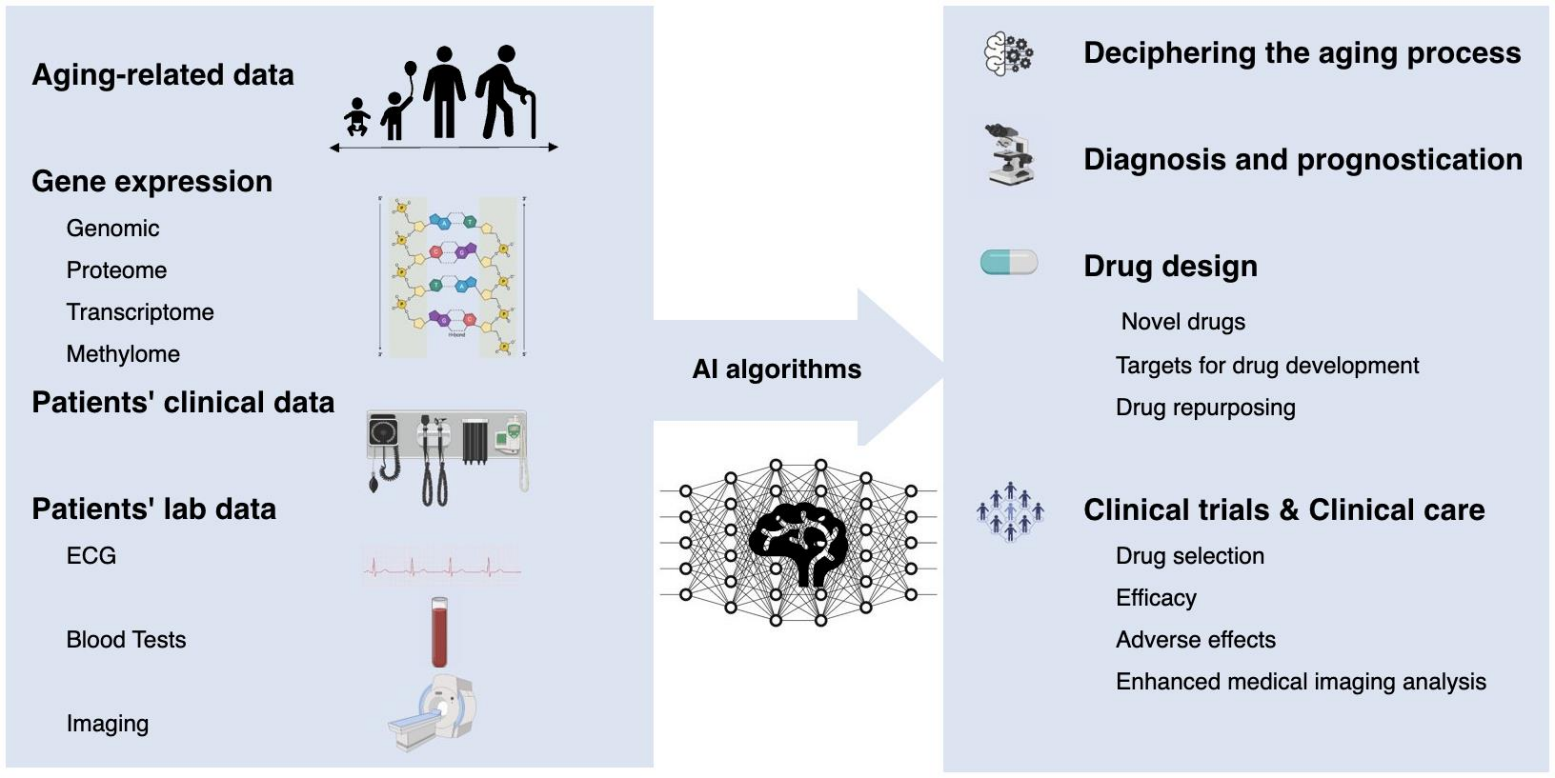
Artificial intelligence for aging research in cancer drug development.

Aging is a multifactorial and complex process associated with various diseases, including cancer. In light of the growing aging population, the need for effective cancer treatments is more significant than ever. Artificial intelligence (AI) is playing an increasingly crucial role in aging research and cancer drug development in revealing the critical drivers of outcomes among a wide range of intrinsic and extrinsic factors [1] (Figure 1).

In recent years, the use of AI in aging research has been rapidly increasing, suggesting that AI-based analysis of healthcare data may enhance clinical care. Herein, we provide an overview of the potential benefits as well as the technical caveats of adopting AI to help researchers identify new targets, develop more effective therapies, and accelerate the discovery process of drug development in the context of aging research.

In this review, we discuss how we could leverage AI technologies to consider a patient’s unique aging profile and tailor cancer treatment more precisely to the individual. This approach would help optimize treatment outcomes, minimize treatment-related risks, and improve the overall quality of care for patients, considering the complex interplay between aging and cancer treatment response.

**Advantages of AI**

AI can identify patterns and relationships not immediately apparent to humans, allowing researchers to develop more effective drugs and therapies. Using machine learning and deep learning, AI can analyze large amounts of data, including genomic data, clinical trial results, and scientific literature, to accelerate cancer drug development.

**Deciphering the aging process**

AI could help identify biomarkers of aging by analyzing vast amounts of genetic and molecular data. Learning more about the aging process through AI would help researchers understand the underlying mechanisms of aging and develop targeted interventions to address them. Moreover, AI would enable researchers to predict the effectiveness of interventions designed to slow down or reverse the aging process. Distinguishing biological pathways of aging in detail could also help investigators design more potent and effective drugs that can target specific molecules [2].

**Cancer diagnosis**

AI algorithms have been demonstrated to be highly effective in predicting cancer diagnosis in the elderly. Machine learning models can study various molecular and epigenetic biomarkers to anticipate the probability of cancer progression in an individual [2]. They can also integrate large amounts of data extracted from patient examinations and analyze them more accurately than clinicians, which reduces the risk of misdiagnosis [1].

**Selection of drug targets for drug development in clinical trials**

Using large-scale data sets, machine learning models can identify novel targets for cancer treatment. These models can be helpful in different aspects of drug design. They can propose and generate novel molecules with desirable molecular and biological features to be used as new drugs [2]. They can also be used to investigate various factors and pathways in regenerative medicine that can effectively control cell deviation cycles and guide them in a specific direction [3]. Gene therapy is another field that can benefit from AI, as it could help researchers choose a specific gene to develop a therapy best suited for an individual patient [2]. AI can also enhance treatment decision and evaluation by quantifying and monitoring changes in combinations of biomarkers [2].

**Drug repurposing**

Several studies have been published on how AI can find new targets or indications for drugs that have been approved for another purpose [4]. AI is able to identify existing drugs that may be effective in treating cancer by analyzing drug databases and molecular interactions.

**Therapeutic dose optimization**

Aging can affect drug metabolism and clearance, potentially impacting the optimal dosage for cancer treatment. AI algorithms can analyze patient-specific factors such as age, renal function, liver function, and medication history to estimate appropriate drug doses. By considering the patient’s unique aging profile, AI can assist in optimizing therapeutic dosages to maximize treatment effectiveness while minimizing the risk of adverse effects.

**Prediction of drug efficacy**

Machine learning models have additionally been trained to predict the success of a drug candidate. Evaluating the efficacy of novel drug regimens and their impact on a patient’s quality of life is crucial. One obstacle in studying aging is that it may take decades for researchers to collect data on a cohort of patients, as aging is a long-term process confounded by environmental and financial factors [2]. Once such datasets are created, AI tools could predict expected treatment response, side effects, and complications based on a patient’s aging profile. This could help healthcare providers adjust treatment plans and provide timely interventions to prevent adverse outcomes. AI could more precisely predict disease recurrence and survival time based on enormous multifactor databases comprising a wide range of patients [1].

**Patient-specific drug selection**

By analyzing patient data, AI could help identify patient-specific treatment options and potential adverse reactions based on molecular and genetic markers. AI tools could also help determine the most optimal treatment regimen for older patients with cancer based on their unique medical history and genetic profile. These personalized treatment plans would likely be more effective and cause fewer side effects. Moreover, enhanced prediction of disease outcome and risk of recurrence would significantly advance precision medicine and personalized cancer care.

**Clinical trial optimization**

AI can improve the efficiency and success rates of clinical trials by identifying eligible patients, stratifying participants based on molecular characteristics, and predicting treatment response. AI algorithms can also optimize trial design, identify potential risks and benefits, and facilitate the selection of appropriate endpoints, resulting in more targeted and efficient clinical trials [2, 3].

**Prediction of adverse events**

Studying the adverse effects of newly designed drugs is essential, as such effects can have a substantial and irrevocable impact on patient’s lives. Recent AI tools have been designed to search through vast amounts of data and estimate the adverse effects of new drugs based on their similarities (e.g., molecular and structural) to existing compounds that have been used to treat the same or similar conditions [5].

**Enhanced medical imaging analysis**

AI algorithms can analyze medical images such as CT scans, MRIs, and PET scans to improve the accuracy of cancer diagnosis and to enhance treatment planning, response evaluation, and prediction of outcome as well as side effects. These methods can use images exclusively or in combination with other medical data to enhance patient management [2]. AI has emerged as a promising tool to address a wide range of clinical challenges, including drug development, clinical diagnosis, treatment selection, clinical trial design, and prediction of patient outcomes [6–8].

**Conclusions**

AI could enhance cancer care and lead to personalized treatment based on age, among other patient-specific characteristics. By taking into account patients’ aging profiles, including physiological changes, comorbidities, and treatment tolerability, AI predictive models can provide evidence-based recommendations, considering factors such as potential drug interactions and the impact of aging on drug metabolism and toxicity. This is a major advancement that can help clinicians make more informed decisions and optimize treatment approaches for individual patients.
